# Observing protein dynamics during DNA-lesion bypass by the replisome

**DOI:** 10.3389/fmolb.2022.968424

**Published:** 2022-09-21

**Authors:** Elise M. Wilkinson, Lisanne M. Spenkelink, Antoine M. van Oijen

**Affiliations:** ^1^ Molecular Horizons and School of Chemistry and Molecular Bioscience, University of Wollongong, Wollongong, NSW, Australia; ^2^ Illawarra Health and Medical Research Institute, Wollongong, NSW, Australia

**Keywords:** DNA replication, protein dynamics, single-molecule, cryo-EM, cyclobutane pyrimidine dimer, lesion bypass, translesion synthesis

## Abstract

Faithful DNA replication is essential for all life. A multi-protein complex called the replisome contains all the enzymatic activities required to facilitate DNA replication, including unwinding parental DNA and synthesizing two identical daughter molecules. Faithful DNA replication can be challenged by both intrinsic and extrinsic factors, which can result in roadblocks to replication, causing incomplete replication, genomic instability, and an increased mutational load. This increased mutational load can ultimately lead to a number of diseases, a notable example being cancer. A key example of a roadblock to replication is chemical modifications in the DNA caused by exposure to ultraviolet light. Protein dynamics are thought to play a crucial role to the molecular pathways that occur in the presence of such DNA lesions, including potential damage bypass. Therefore, many assays have been developed to study these dynamics. In this review, we discuss three methods that can be used to study protein dynamics during replisome–lesion encounters in replication reactions reconstituted from purified proteins. Specifically, we focus on ensemble biochemical assays, single-molecule fluorescence, and cryo-electron microscopy. We discuss two key model DNA replication systems, derived from *Escherichia coli* and *Saccharomyces cerevisiae*. The main methods of choice to study replication over the last decades have involved biochemical assays that rely on ensemble averaging. While these assays do not provide a direct readout of protein dynamics, they can often be inferred. More recently, single-molecule techniques including single-molecule fluorescence microscopy have been used to visualize replisomes encountering lesions in real time. In these experiments, individual proteins can be fluorescently labeled in order to observe the dynamics of specific proteins during DNA replication. Finally, cryo-electron microscopy can provide detailed structures of individual replisome components, which allows functional data to be interpreted in a structural context. While classic cryo-electron microscopy approaches provide static information, recent developments such as time-resolved cryo-electron microscopy help to bridge the gap between static structures and dynamic single-molecule techniques by visualizing sequential steps in biochemical pathways. In combination, these techniques will be capable of visualizing DNA replication and lesion encounter dynamics in real time, whilst observing the structural changes that facilitate these dynamics.

## Introduction

DNA replication is carried out by the replisome, a multi-protein complex responsible for the coordination of DNA unwinding and synthesis on both daughter strands. Correct and complete DNA replication faces challenges of both intrinsic and extrinsic nature (Tiwari and Wilson, 2019). Intrinsic processes that result in physical roadblocks to DNA replication include DNA transcription, repair, and any process that involves DNA-binding proteins, as well as intrinsic oxidative processes which can lead to oxidative-stress related lesions (Pizzino et al., 2017). Extrinsic challenges can arise from DNA-damaging agents, such as chemicals and ultraviolet (UV) radiation.

UV radiation can cause DNA damage in the form of UV-induced DNA lesions. Intracellular DNA repair mechanisms have evolved that can repair these lesions. However these repair mechanisms are not always successful. Should the replisome encounter these lesions, replication will be stalled or otherwise impaired. This disruption to DNA replication can lead to genomic instability, which, in humans, may contribute to the development of cancers, such as skin cancer (Gaillard et al., 2015).

The replisome can bypass these unrepaired lesions in an attempt to prevent fork collapse and replisome halting, however this is often highly mutagenic in nature, and therefore still contributes to genomic instability. Lesion bypass is facilitated by DNA damage tolerance (DDT) pathways, such as translesion synthesis (TLS), in which specialized TLS polymerases are recruited to synthesize DNA across the lesions in an error-prone way (Bi, 2015).

The replisome is a multi-protein complex with its stability determined by an intricate network of pairwise protein–protein and protein–DNA interactions (Scherr et al., 2017). Due to this multitude of interactions, proteins can dynamically interact with the replisome, binding and unbinding at rates depending on the conditions (Mueller et al., 2019; Lewis et al., 2020). The effect that these dynamics have on the outcome of encountering a lesion has been the interest of many studies.

Both bacterial and, more recently, eukaryotic replisomes can now be fully reconstituted from purified proteins. With these reconstituted systems, experiments to probe protein dynamics can be done *in vitro*. With precise control over experimental conditions, such as protein concentration and buffer conditions, *in vitro* experiments can provide a detailed characterization of molecular pathways (Tanner et al., 2009).

Classical biochemical assays have long been the method of choice to study DNA replication. These assays, that average readouts over large ensembles of molecules, have already provided much insight into lesion bypass. For more detailed kinetic information, single-molecule techniques have been established. A single-molecule approach allows for the visualization of molecular processes and properties at the single-molecule level. This allows for the characterization of subpopulations, visualization of transient intermediates, and the collection of detailed kinetic information. In addition to ensemble and single-molecule microscopy techniques, Cryo-EM can also provide insight into protein dynamics and lesion bypass by obtaining detailed structures of proteins and complexes involved. This highlights structure flexibility and therefore any potential dynamics that individual subunits within the replisome may be capable of.

This review will focus on these established approaches for the *in vitro* study of the dynamic behavior of replisome proteins during lesion bypass, using UV-induced DNA lesions as a key example. We will discuss the key benefits and challenges associated with each of these techniques, including how these methods can help us answer key questions about lesion bypass. These key questions include: how do dynamics play a role in lesion bypass and what is the role of TLS polymerases? In addition, we will highlight opportunities for future research.

## Chemical DNA lesions as a roadblock for DNA replication

### The replisome and model systems for DNA replication

**FIGURE 1:**
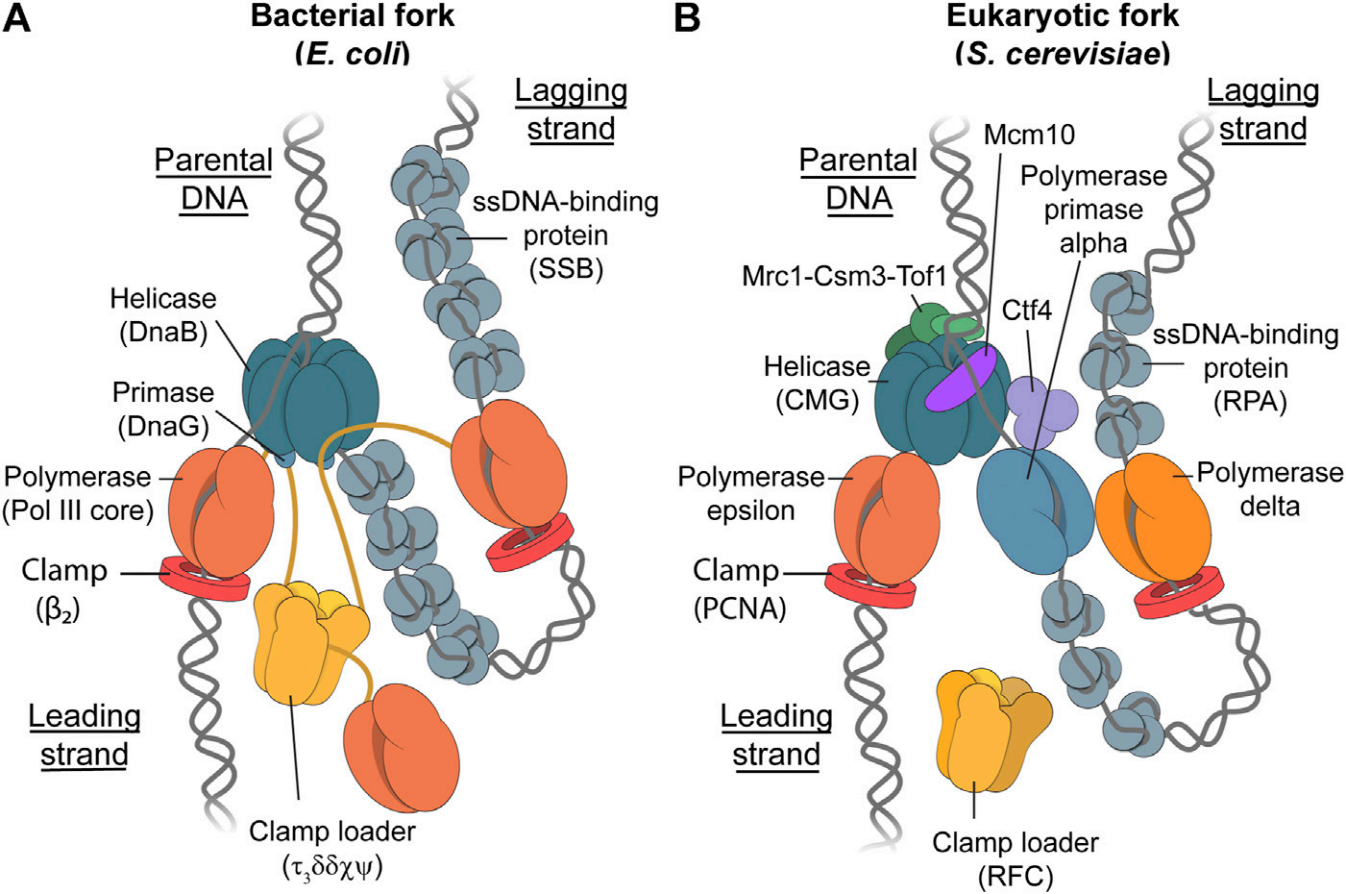
The Bacterial and Eukaryotic Replisomes. Schematic representations depicting the bacterial replisome (Stamford et al., 1992; Oakley et al., 2003; Tanner et al., 2008; Jergic et al., 2013; Mason et al., 2013) **(A)** and eukaryotic replisome (Bell and Labib, 2016; O’Donnell and Li, 2016; Coster and Diffley, 2017; Georgescu et al., 2017; Miller et al., 2019) **(B)**. While the main components in each replisome fulfill similar roles, the eukaryotic replisome requires more proteins for proper function. These additional proteins include Mcr1-Tof1-Csm3 for controlling replisome speed, Mcm10 for helicase loading and stability, and Ctf4 which likely acts as a protein-binding hub (Villa et al., 2016). Furthermore, the yeast replisome has three different replicative polymerases where prokaryotes only have one. The clamp-loader complex in *E. coli* forms a stable complex with up to three core polymerase complexes to form Pol III*, whereas it is unknown whether the RFC clamp loader in yeast travels with the fork or binds transiently.

Genomic integrity relies on the faithful replication of the entire genome. The replication of DNA is carried out by a multi-protein complex called the replisome. The general composition of the replisome is conserved across all domains of life, with the key replisomal proteins being: the *helicase*, which is required for the efficient unwinding of double-stranded DNA (dsDNA) to create two single-stranded DNA (ssDNA) templates; *DNA polymerases* which are responsible for synthesizing new DNA strands on the previously unwound ssDNA template; the *primase* which primes the leading and lagging DNA strands in order to initiate DNA synthesis; and *single-stranded DNA-binding proteins (SSBs)* which are responsible for the stabilization of ssDNA through binding, as well as regulating DNA replication, recombination, and repair (Figure 1) (Sun B et al., 2015).

Common model systems for DNA replication include *Escherichia coli* (*E. coli*) for the study of bacterial DNA replication (Figure 1A), and budding yeast (*Saccharomyces cerevisiae*) as a model for eukaryotic DNA replication (Figure 1B). *E. coli* is often used a model system for bacterial DNA replication due to its ease and speed of growth, as well as it being one of the earliest genome sequences to be published (Blattner et al., 1997). These factors have led to this system becoming the most well-studied replisomal system in literature (Lewis et al., 2016). Budding yeast (*Saccharomyces cerevisiae*) is of particular interest for the modeling of eukaryotic DNA replication. Not only is the composition of the yeast replisome similar to the human replisome (Guilliam and Yeeles, 2021), cryo-EM studies showed that the general architecture of the yeast replisome is very similar to the human replisome (Bai et al., 2017; Jones et al., 2021). Furthermore, yeast was the first eukaryote to have its genome completely sequenced and published in 1997 (Botstein et al., 1997). In 2015, a functional eukaryotic replisome was reconstituted from purified proteins in an *in vitro* study for the first time (Georgescu et al., 2015; Yeeles et al., 2015). This system consists of 31 distinct polypeptides and is capable of both lagging-strand and leading-strand synthesis.

### Genomic instability

Incomplete or incorrect DNA replication can lead to genomic instability. Genomic instability can be induced by replication stress, including any obstacle to DNA replication. Genomic instability can range from elevated base-pair mutation counts, all the way to significant structural abnormalities such as variations in chromosome count or structure (Yao and Dai, 2014). These instabilities can lead to a number of diseases, some of which are associated with neurodegeneration, immunodeficiencies, intellectual disabilities, and UV-light sensitivity. One of the most notable diseases associated with genomic instability is cancer, with genomic instability being a characteristic of almost all human cancers (Negrini et al., 2010). Population cancer statistics highlight the seriousness of genomic instability, with approximately 39.5% of men and women being diagnosed with some form of cancer in their lifetime in the United States (National Cancer Institute, 2020).

### Ultraviolet-radiation exposure can cause genomic instability

While genomic instability can cause disease, it can also be caused by a disease. In some cases, genomic instability can have heredity causes, with various syndromes, such as Lynch syndrome, causing mutations in DNA repair genes, leading to a high replicative stress (Negrini et al., 2010).

**FIGURE 2:**
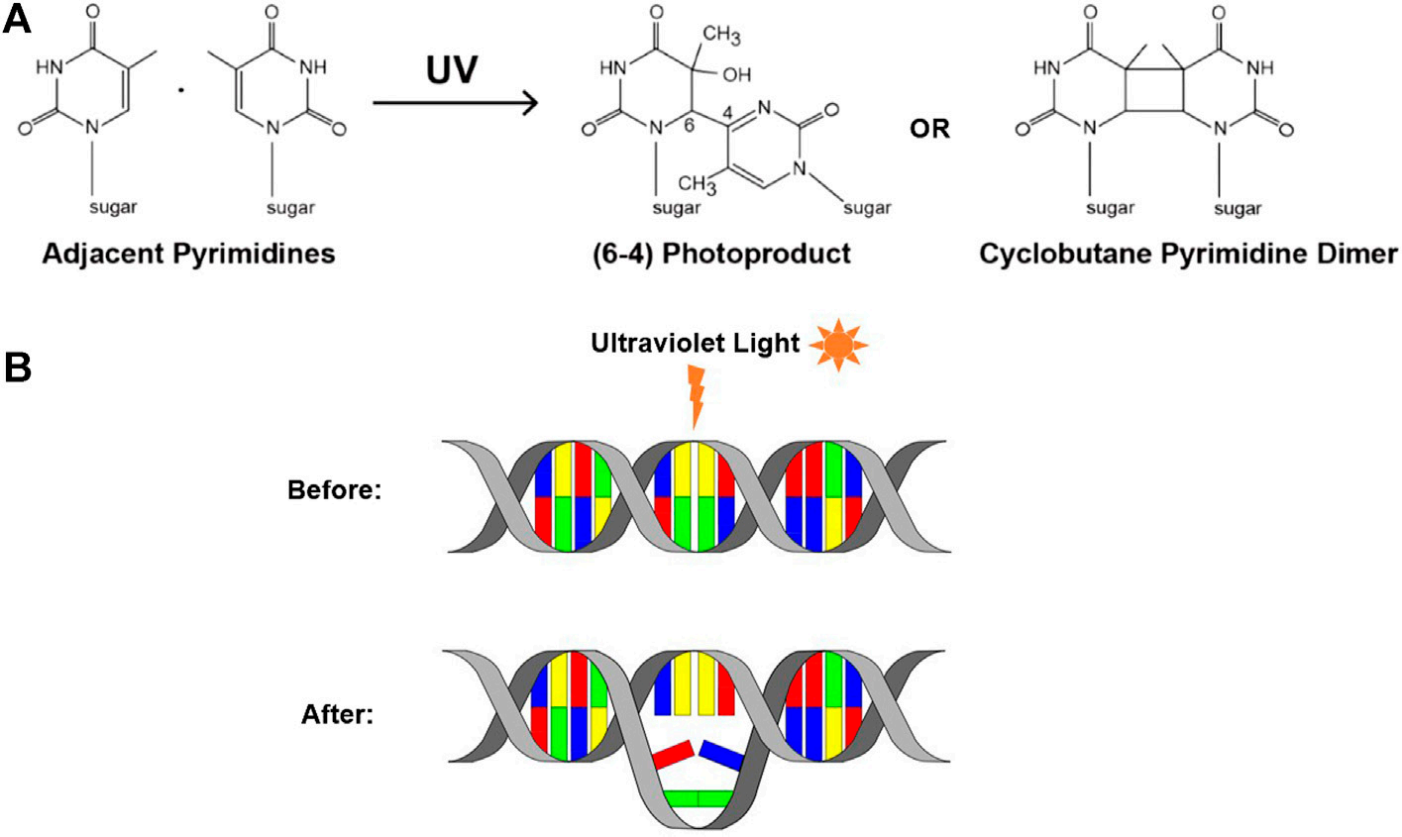
UV-induced DNA lesions. **(A)** UV exposure can cause neighboring pyrimidine bases to form covalent bonds, resulting in one of two UV-induced lesions: a 6-4PP or a CPD (adapted from Atdbio, 2021). **(B)** Schematic depicting the DNA distortion that these lesions can cause.

However, a notable and extremely common cause of genomic instability is exposure to UV radiation. Exposure to UV radiation causes DNA damage in the form of UV-induced DNA lesions. UV radiation falls between the wavelengths of 100 and 400nm, and can be further divided into UV-A, UV-B, and UV-C (Fu, 2002). While UV-C is the most dangerous of the three, DNA damage is largely caused by UV-A and UV-B, due to absorption by atmospheric ozone (Vehniäinen et al., 2012). The DNA damage that is induced by UV-A and UV-B radiation manifests itself in the form of dimeric photoproducts. Upon UV exposure, adjacent pyrimidine bases on a single strand of DNA can form covalent bonds which form the basis of these disruptive lesions. These dimers can occur in one of two forms, the first is a cyclobutane pyrimidine dimer (CPD), and the second a pyrimidine (6-4) pyrimidone photoproduct (6-4PP) (Mouret et al., 2006) (Figure 2A).

Both of these forms of dimerized bases can distort the shape of DNA and impair the ability of the DNA polymerases to incorporate the nucleotides, correct or otherwise, opposite the lesion (McCulloch et al., 2004) (Figure 2B). Consequently, this can cause the replisome to stall or collapse, and therefore represents replication stress. It is important to note that, while these lesions can lead to the development of mutations within DNA, they are not mutations themselves. Instead, they are a form of premutagenic DNA damage. Due to their frequency and prevalence, these UV lesions are a notable example of a chemical roadblock to DNA replication, and can be used to investigate the replisome’s response to encountering chemical roadblocks.

### Repair of ultraviolet-induced lesions

Despite the prevalence of UV-induced lesions, mutations and subsequent complications can be prevented through DNA-repair pathways. In many organisms, prokaryotic and eukaryotic, resistance to UV radiation increases when UV exposure is closely followed by exposure to visible light (Sancar, 1994). This is called photoreactivation and involves enzymes called photolyases, which utilize the energy of visible light to break the cyclobutane ring of UV dimers. This results in two intact monomers and DNA with restored integrity (McCready and Marcello, 2003). Due to structural differences, photolyases are specific to either CPDs or 6-4PPs, meaning that some organisms possess the ability to photoreactivate both CPDs and 6-4PPs, and others only one of the two.

Despite this mechanism of repair being widespread amongst most organisms, it is completely absent in placental mammals. Humans and other placental mammals therefore rely on a pathway called nucleotide excision repair (NER) in order to repair UV lesions and prevent mutations from occurring. NER works by incising the DNA strand either side of the dimer and then removing damaged DNA in the form of an oligomer. This leaves behind a ssDNA gap that is then filled in through “repair synthesis”, followed by ligation. This ultimately results in the restoration of the undamaged dsDNA (Huang et al., 1992). In *E. coli*, NER requires just six proteins, however mammalian NER is much more complex, with roughly thirty proteins being required (Aboussekhra et al., 1995). These thirty proteins include proteins that are utilized in normal DNA metabolism and replication, as well as nine major NER proteins: XPA, XPB, XPC, XPD, XPE, XPF, XPG, CSA, and CSB (de Boer and Hoeijmakers, 2000).

The mutagenic potential of pyrimidine dimers can be highlighted by a rare genetic disorder called Xeroderma Pigmentosum (XP). A defect in one or more of these *Xeroderma Pigmentosa* genes (XPA-XPG) would result in a reduced efficiency of the NER pathway, often resulting in UV-induced DNA damage being left unrepaired (Niedernhofer et al., 2006). An individual with XP would therefore be very sensitive to UV, causing them to accumulate CPDs, 6-4PPs and resultant mutations throughout their lifetime. This accumulation which would almost certainly lead to the development of skin cancer, as well as serious neurological symptoms (Hengge and Emmert, 2008). If no extreme intervention is taken, such as avoiding sunlight all together, then it is expected that those with the disorder may only have a life expectancy of between 20 and 30 years. The failure of NER that characterizes this rare disorder presents an extreme example of the detrimental effects of genomic instability, as well as highlighting the importance of repairing roadblocks before replisome encounter.

When the replisome encounters a lesion before repair. Replication may pause or cease, which can have serious consequences for the replisome, including fork collapse. In some cases, lesions can be bypassed in a mutagenic manner in order to prevent replication halting, and to prevent fork collapse (Hedglin and Benkovic, 2017). This error-prone bypass is a worthwhile compromise to the cell as incorrect nucleotide insertion is preferable to fork collapse and incomplete replication, despite still contributing to genomic instability (Rizzo and Korzhnrv, 2019).

## Protein dynamics provide pathways for lesion bypass

All mechanisms of lesion bypass rely on a dynamic replisome capable of protein exchange (Mueller et al., 2019). Specifically, replicative polymerases are generally unable to synthesize across a lesion. As a result, the polymerase will stall at the site of the lesion. Through exchange of the stalled polymerase, continued replication can be ensured. There are two main mechanisms proposed for this process: replicative translesion synthesis (TLS) and lesion skipping.

### Replicative translesion synthesis

TLS has been proposed to mainly play a role upon encountering leading-strand lesions. This process is facilitated by specialized DNA polymerases that belong to the Y-family (Yang, 2014). There are over 300 enzymes in the Y-family, with members identified in bacteria, archaea, and eukaryotes (Yang and Woodgate, 2007). Individual organisms often possess more than one of these Y-family TLS polymerases, with *S. cerevisiae* possessing two; DNA Pol η and Rev1 (Ohmori et al., 2001). In addition to these Y-family polymerases, *S. cerevisiae* also requires DNA Pol ζ, which belongs to the B-family (Johnson et al., 2006). These same TLS polymerases are used in human TLS, along with two additional polymerases: DNA Pol ι and DNA Pol κ. *E. coli* has three TLS polymerases: Pol II, Pol IV, and Pol V (Goodman and Woodgate, 2013).

High-fidelity DNA polymerases used in unimpeded DNA replication, such as Pol δ and Pol ε in the case of the yeast replisome, are not able to efficiently synthesize over lesions as their active site cannot accommodate for the bulky lesion structure. TLS polymerases overcome this issue due to their flexible DNA-binding domains and variable binding pocket, allowing them to accommodate various DNA lesions and, hence, facilitate lesion bypass by the replisome (Yang and Woodgate, 2007).

**FIGURE 3:**
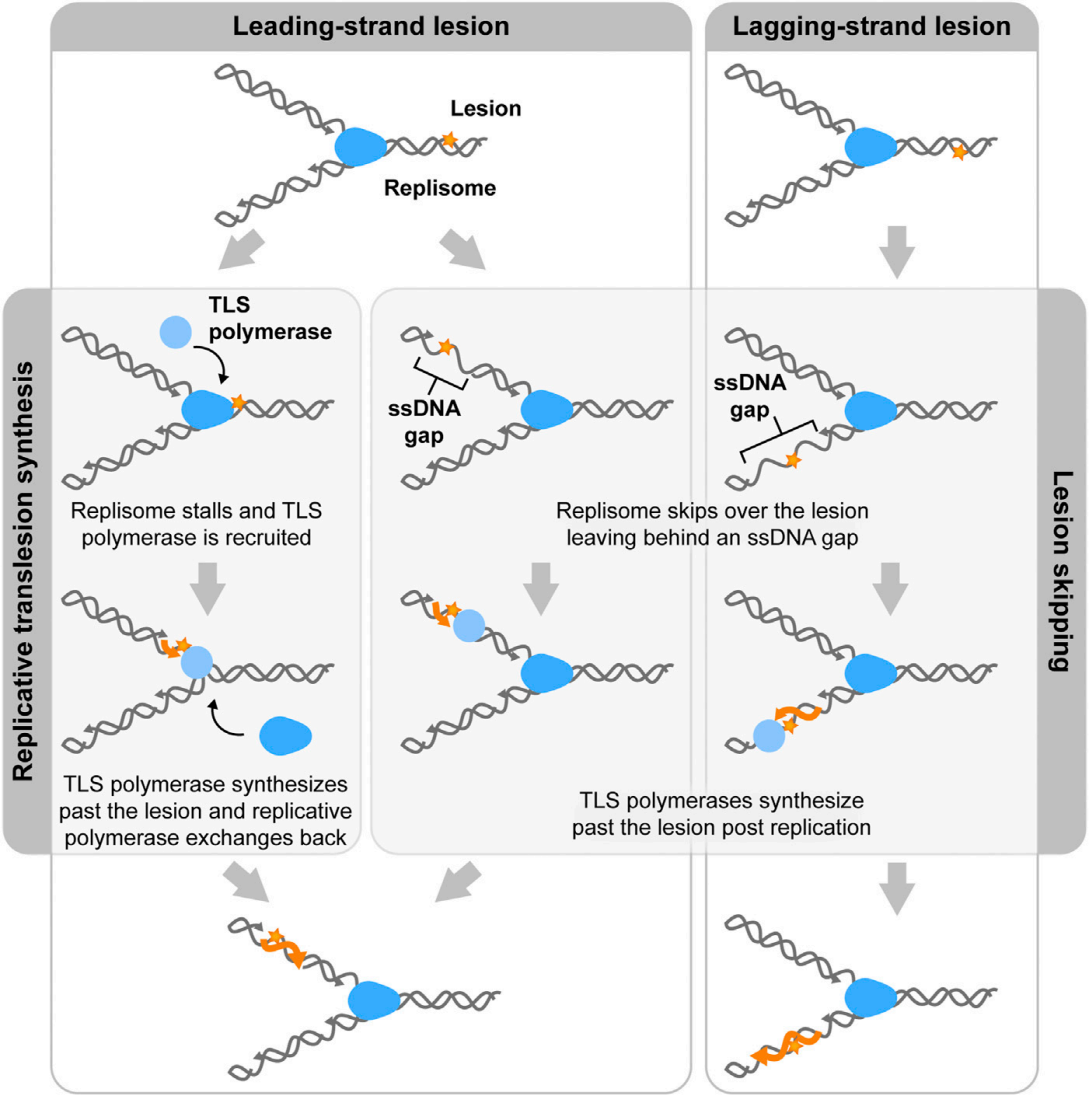
Two mechanisms of lesion bypass. Both bacterial and eukaryotic replisomes can bypass DNA lesions by translesion synthesis (TLS) or lesion skipping. In translesion synthesis, specialized TLS polymerases (light blue) exchange into the replisome (blue) to synthesize over the lesion (orange star). Replicative polymerases then exchange back into the replisome to continue replicating. In the lesion skipping pathway, the replisome moves past the lesion and reinitiates replication downstream, leaving a lesion containing ssDNA gap in the wake of the replisome. This gap is then later filled by TLS polymerases.

In replicative TLS, these TLS polymerases are recruited in the presence of damaged DNA (Figure 3, left). In the eukaryotic replisome, when DNA is damaged, high-fidelity polymerases stall and the PCNA clamp, which is responsible for providing binding sites for the polymerases, is mono-ubiquitylated. This facilitates exchange of the replicative polymerase for a TLS polymerase and retention of TLS polymerase to the damaged sites (Boehm et al., 2016; Lancey et al., 2021). These dynamics must be tightly regulated due to the low-fidelity of TLS polymerases. The recruitment of these TLS polymerases is tightly regulated to prevent them from copying undamaged DNA (Rizzo and Korzhnrv, 2019).

A similar mechanism has been proposed for TLS in *E. coli* (Heltzel et al., 2012; Fuchs and Fujii, 2013; Scotland et al., 2015). Upon stalling of the replicative polymerase, TLS polymerases exchange into the replisome through binding to the β_2_ clamp. This model was primarily built upon the results of *in vitro* reconstitution assays and led to the proposal of molecular mechanisms invoking polymerase switching on the β clamp (Wagner et al., 2000; Becherel et al., 2002; Lenne-Samuel et al., 2002; Furukohri et al., 2008; Kath et al., 2014; Kath et al., 2016). This mechanism was proposed mainly based on *in vitro* experiments. However, *in vivo* single-molecule experiments have shown that TLS polymerases mainly act away from replisomes (Robinson et al., 2015; Thrall et al., 2017; Henrikus et al., 2018a; Henrikus et al., 2018b). Therefore, replicative TLS does not seem to be a dominant pathway for replisome bypass of lesions in bacteria.

### Lesion skipping

The second proposed mechanism for bypass, lesion skipping, involves the replisome moving past the lesion and the reinitiation of synthesis downstream of the lesion (Figure 3, middle, right). In the case of lagging-strand lesion skipping, the replisome terminates the nascent Okazaki fragment upon encountering the lesion. The replicative helicase can accommodate the lesion, allowing for DNA unwinding to occur past the lesion. Lagging-strand synthesis can then be reinitiated through the normal Okazaki-fragment priming mechanism. The Okazaki fragment that contains the lesion is left behind as an unreplicated ssDNA region. TLS polymerases can replicate over the lesion outside of the context of the replisome. Lagging-strand lesion skipping has previously been shown to be rapid and efficient (Higuchi et al., 2003; McInerney and O'Donnell, 2004; Taylor and Yeeles, 2018). Leading-strand lesion bypass is a harder to imagine, as replication restart has to involve repriming of the leading strand (Figure 3, middle). Studies on both the bacterial and eukaryotic replisomes have shown evidence of leading-strand lesion bypass (Gabbai et al., 2014). However, this process has been shown to be less efficient than lagging-strand lesion skipping. (Yeeles and Marians, 2013; Gabbai et al., 2014).

A key question is what happens to the polymerases upon lesion skipping. It has been suggested that the polymerase unbinds from the lesion-containing DNA template and remains bound to the helicase. Once a new primer is synthesized this polymerase can re-engage with the DNA to continue synthesis. Recently it has been shown that, under conditions of unimpeded replication, replicative polymerases are rapidly exchanging with other replicative polymerases in the environment (Loparo et al., 2011; Geertsema et al., 2014; Beattie et al., 2017; Lewis et al., 2017; Kapadia et al., 2020; Lewis et al., 2020). This observation provides an alternative mechanism for what happens to the polymerases upon lesion skipping. Once a polymerase encounters a lesion, the stalled polymerase decouples from the helicase (Graham et al., 2017) and is left behind at the lesion. A new polymerase from solution will exchange into the replisome to occupy the vacant spot left by the original polymerase. Once a new primer is synthesized, a new polymerase can exchange into the replisome to resume synthesis.

## Ensemble studies

For over three decades ensemble biochemical assays have been used to probe protein dynamics during lesion bypass by replisomal proteins (Tippin et al., 2004; Branzei and Foiani, 2005; Langston and O’Donnell, 2006; Yeeles et al., 2013; Marians, 2018). While these techniques do not have the spatiotemporal resolution to directly monitor the behavior of individual proteins, protein dynamics can still be derived. Here we highlight studies from which protein dynamics have been inferred.

Already in 1989 it was shown that the T7 helicase, which is responsible for unwinding parental DNA within the bacteriophage T7 replisome, is blocked by a single DNA lesion through the use of a nucleotide hydrolysis assay (Brown and Romano, 1989). Nucleotide hydrolysis by the helicase was fully inhibited by the presence of a lesion on the DNA template. This observation not only showed that the helicase is unable to unwind past the lesion, but also inferred that the stalled helicase remained stably bound to the DNA template.

**FIGURE 4:**
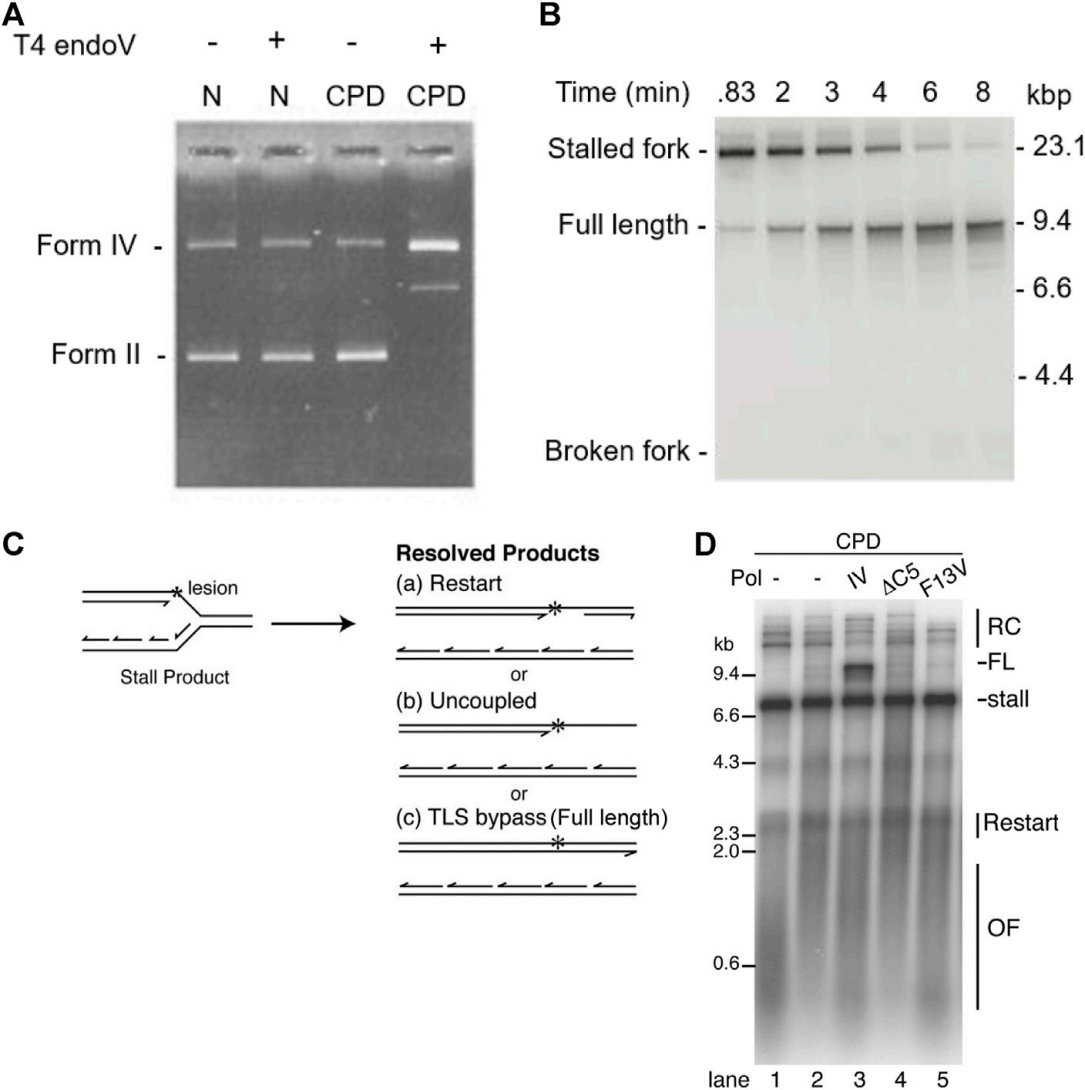
Ensemble methods: gel electrophoresis. **(A)** UV exposed DNA was treated with T4 endonuclease V and then analyzed using gel electrophoresis. In the absence of UV exposure, T4 endonuclease V had no effect on the position or intensity of the bands. In the presence of UV exposure, T4 endonuclease V nicked the DNA at CPD sites, causing a change of form and intensity of the bands and, hence, confirming presence of CPDs (Carty et al., 1996). **(B)** Time course experiment showing the *E. coli* replisome can bypass a CPD lesion and reinitiate replication downstream of the lesion (Yeeles and Marians, 2013). **(C)** replication reaction scheme showing the possible replicated DNA products. **(D)** Replisome-mediated Pol IV-catalyzed TLS bypass requires interaction with β_2_ and TLS activity, suggesting that replicative polymerases can dynamically exchange (Gabbai et al., 2014).

In 1996, Carty el al. (1996) treated exposed DNA with T4 endonuclease V, which nicks at UV-induced DNA lesions. Conversion of closed circular DNA to nicked circular DNA was detected as a change to slower mobility by UV transillumination and photography (Carty et al., 1996) (Figure 4A). In addition, the exact position of the lesion in the plasmid construct was mapped by primer extension analysis.

Using linear DNA templates with specifically introduced leading-strand CPD lesions and showing progression of DNA synthesis using gel electrophoresis, it was shown that the *E. coli* replisome is able to bypass these lesions and continue replication (Heller and Marians, 2006; Yeeles and Marians, 2013) (Figure 4B). Although a leading-strand lesion may stall the replication fork temporarily, the lesion need not be repaired to resume DNA synthesis. Furthermore, the data suggest that continued template unwinding is likely to be crucial for leading-strand reinitiation, as it ensures sufficient ssDNA on the leading-strand template to enable primer synthesis to occur (Yeeles and Marians, 2013). Again, these data seem to suggest that the helicase remains stably bound as an active helicase. In contrast, the rate of replication reinitiation was found to be dependent on the primase concentration (Nevin et al., 2017). This observation suggests that the primase does not remain stably bound to the helicase, but instead rebinds after the helicase bypasses the lesion. The efficiency of replication restart was shown to be dependent on clamp assembly, suggesting that both the clamp as well as the clamp loader are not stably bound to the replisome. Several ensemble studies show that TLS polymerases can exchange into the replisome upon encountering a lesion (Furukohri et al., 2008; Indiani et al., 2009; Gabbai et al., 2014; Kath et al., 2016). Figures 4C,D shows lesion bypass and replication restart in the absence of TLS polymerases. However the presence of the TLS polymerase Pol IV, results in an additional reaction product corresponding to replicative TLS. The activity of Pol IV in this reaction critically depends on interactions with the β2 clamp. These observations suggest that the replicative polymerase can behave in a dynamic fashion, unbinding and rebinding to the replisome.

The eukaryotic replisome is similarly able to bypass DNA lesions and reinitiate replication past the lesion. Reinitiation of leading-strand synthesis was shown to be promoted by RPA depletion suggesting a dynamic equilibrium between RPA and the polymerase-primase Pol α (Taylor and Yeeles, 2018). Following helicase-polymerase uncoupling, a switch from Pol ε, the canonical leading-strand polymerase, to the lagging-strand polymerase Pol δ, facilitates rapid and efficient lesion bypass (Guilliam and Yeeles, 2021).

Methods based on gel electrophoresis to study the effect of roadblocks can be expanded to 2D gels (Mettrick and Grainge, 2016). Here, DNA products are run on an agarose gel as in a regular gel electrophoresis assay, before running each lane in perpendicular direction (Friedman and Brewer, 1995; Courcelle et al., 2003). 2D gels allow for the separation of branched DNA structures. As such, the technique can be used to observe UV-induced intermediates associated replication arrest. Using these 2D gels and thermosensitive mutants is was suggested that the *E. coli* polymerases can transiently dissociate from the DNA upon encountering a lesion (Kunzelmann et al., 2010). In contrast, the helicase–primase complex seemed to remain stably associated with the DNA.

Other ensemble studies with different objectives exist, such as Courcelle et al., 2003, where *E. coli* DNA is exposed to UV radiation in order to observe transient inhibition and its subsequent recovery. In this study, 2D gel electrophoresis is used to visualize the replication intermediates by first purifying at various times after UV irradiation, and then digesting with an enzyme that cuts the plasmid just downstream of the unidirectional origin of replication. This creates a migration pattern of replicating molecules. As little is known about the structural characteristics of the intermediates involved in replication recovery, this migration pattern of replicating molecules allowed for the differentiation and identification of the structural properties of these fragments. Hence, insight into replication dynamics was able to be obtained.

These ensemble-averaging methods are highly informative for the purpose many of the studies describe here, as it can be observed whether replication was complete or incomplete in the presence of lesions. Furthermore, most of these methods are relatively easy to carry out and use equipment that can be acquired at comparatively low cost. However, it is challenging using these assays to obtain detailed kinetic or dynamic information. It is this kinetic information that is required in order to help answer the key questions regarding lesion bypass, such as the dynamics of individual proteins, the difference between leading-strand and lagging-strand lesion bypass, and how the proteins that facilitate bypass are recruited. Instead, single-molecule techniques, such as total internal reflection fluorescence (TIRF) microscopy, can be used to visualize DNA replication in real time.

## Single-molecule *in vitro* methods

### Single-molecule approaches provide temporal information

Observing molecular properties at the single-molecule level allows for the characterization of subpopulations and the visualization of rare transient intermediates, heterogeneity, and rapid kinetics. This type of information is hidden by the ensemble-averaging nature of traditional biochemical assays. Unlike ensemble techniques, single-molecule techniques can provide temporal detail: insight into binding events, replication stalling and reinitiation, changes in replication rate, as well as which proteins played a part in these kinetic processes (Stracy et al., 2014). Force-based single-molecule approaches interrogate the changes in the energy landscape during biochemical processes (Monachino et al., 2017). These force-based single-molecule methods have already proven to be a useful tool to study lesion bypass (Fu et al., 2011; Kath et al., 2014; Sun J et al., 2015; Kath et al., 2016; Gahlon et al., 2017). In these studies, optical or magnetic tweezers are used to monitor changes in DNA length upon bypass of the lesion. However, these assays typically do not provide a direct readout of protein binding and unbinding dynamics.

Therefore, in this review, we mainly focus on single-molecule studies using fluorescence. Fluorescence is particularly useful to visualize protein dynamics, as these techniques allow for the direct visualization of the behavior of individual proteins. In fluorescence-based assays, a protein of interest is labeled with a fluorescent molecule.

**FIGURE 5:**
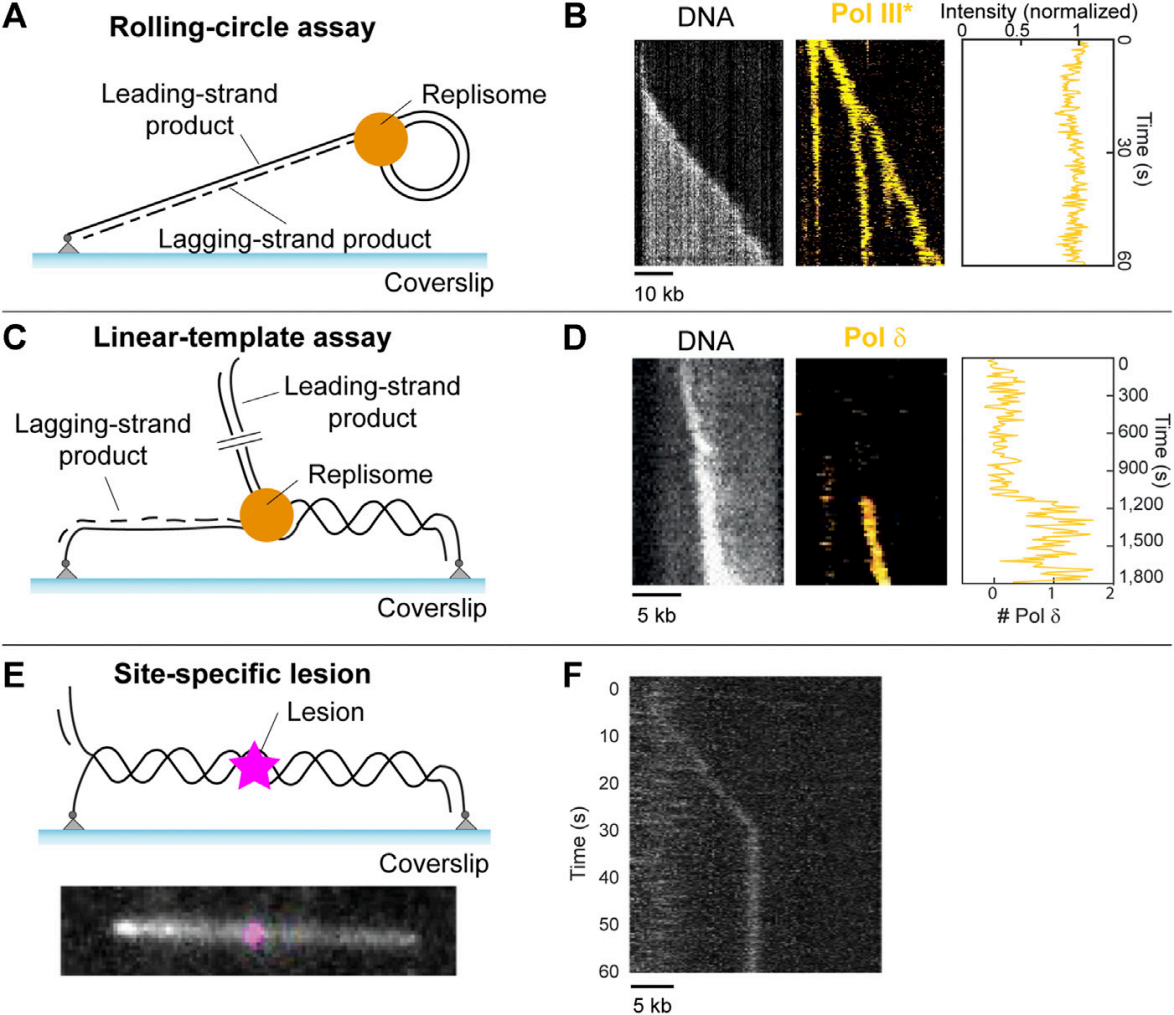
Single-molecule methods: TIRF microscopy. **(A)** Schematic representation of the single-molecule rolling-circle assay. **(B)** Example kymograph of a single molecule showing rolling-circle replication (left, gray). Example kymograph showing the fluorescence of fluorescently labeled polymerases during rolling-circle replication (middle, yellow), and the quantification of the intensity of the polymerases at the fork (right). (Middle and right are adapted from Lewis et al., 2017). **(C)** Schematic representation of the single-molecule linear template DNA replication assay **(D)** Example kymograph depicting visualization of DNA during replication (left, gray), the fluorescence signal from a fluorescently labeled polymerase exchanging into the replisome (middle, yellow), and the quantification of the number of labeled polymerases over time (right) (adapted from Lewis et al., 2020). **(E)** (top) Schematic representation of a 36-kb linear template with a site-specific lesion (magenta) in the middle. (bottom) Example showing a 36-kb linear template, with a Cy5 fluorophore modelling the position of a site-specific roadblock which can be inserted by the same method. **(F)** Example kymograph showing stalling of DNA replication by the *E. coli* replisome at the site of a CPD lesion (adapted from Kaur et al., 2022).

In TIRF microscopy, a ∼100-nm thin layer of aqueous solution near a planar glass substrate can be selectively illuminated, allowing for the dynamics of individual molecules to be studied over time (Axelrod et al., 1984). The fluorescence from these molecules can then be detected using a camera, to create a molecular movie. Factors such as replication efficiency, processivity, and replication rate, as well as any molecule kinetics can be directly extracted from these movies (Figure 5A).

### Rolling-circle assay

In 2009, Tanner et al. developed a TIRF microscopy method that used a rolling-circle DNA template anchored to a surface, allowing for the visualization of single DNA molecules undergoing replication in real time. Reaction components were loaded, and DNA was stretched under flow to allow for the observation of the time-dependent length of the replicating DNA. In 2016, Leisle et al. (2016) further developed this technique to include fluorescently labeled proteins which allow for the visualization of individual replication components during replication.

This development lead to the discovery that polymerases exchange rapidly with proteins from solution during DNA replication (Loparo et al., 2011; Geertsema et al., 2014; Lewis et al., 2017). This went against the previously held belief that polymerases remain stably bound within the replisome during replication, which was assumed for decades prior (Figure 5B). Additionally, it was found that *E.coli* ssDNA binding proteins (SSBs), which are responsible for protecting ssDNA and play a role in replication regulation (Figure 1, left), can remain stably bound within the replisome during replication (Spenkelink et al., 2019). This goes against the previous assumption that SSBs exchange rapidly within the replisome. It was found that the stability of polymerases and SSBs within the replisome depends on the concentration of proteins in solution, that is, it uses a concentration-dependent exchange mechanism (Gibb et al., 2014). Since this mechanism allows the replisome to adapt to its environment, it was hypothesized that this adaptability plays a crucial role in lesion encounter and potential bypass (Spenkelink et al., 2019).

The rolling-circle assay has proven very useful to study replication-roadblock encounters in ensemble assays (Yao et al., 2009; Wang et al., 2014; Killelea et al., 2019; Whinn et al., 2019). However, the lack of spatial resolution between replisome and roadblock makes this template less suitable for single-molecule studies of lesion encounters. In order to determine the effect of roadblocks on DNA replication, clear separation between replisome and roadblock is preferable. Increasing the size of the rolling-circle template could achieve this separation. The size of the template would need to be increased such that the lesion can be spaced away from the replisome at least twice as far as the diffraction limit.

### Linear templates

Another way to provide spatial separation between replisome and lesion, is through the use of linear DNA templates (Kaur et al., 2022).

In 2020, Lewis et al. (2020). described a forked doubly-tethered linear DNA substrate used to visualize DNA replication by a reconstituted eukaryotic replisome at the single-molecule level for the first time. This assay can be seen in Figure 5C, with examples of results obtained from such studies shown in Figure 5D. These results include a kymograph on the left, which is a 2D representation of a molecular movie showing DNA replication of a single molecule as a function of time. These movies are obtained through the use of TIRF microscopy. The DNA is stained with the intercalating dye SYTOX orange. Since SYTOX orange exclusively stains dsDNA, newly synthesized leading-strand DNA appears as a bright spot that increases in intensity with time while moving in a unidirectional manner. Fluorescently labeled replisome proteins can be monitored simultaneously, using two-color imaging (Figure 5D, middle). The assay can be combined with single-molecule fluorescence-recovery-after-photobleaching (FRAP) assays, which can be used to measure the appearance of fluorescence as a result of molecular exchange (Spinks et al., 2021). In these assays, all fluorophores in a field of view are deliberately photobleached using a short high-intensity laser pulse. The recovery of any fluorescence signal indicates that unbleached molecules from solution are able to exchange into the replisome. FRAP therefore allows for the measurement of the dynamic behavior of labeled proteins (Figure 5D, middle, right). This assay was used to show that, similar to the bacterial replisome, polymerases in the yeast replisome exchange with polymerases from solution in a concentration-dependent manner. Furthermore, the assay found evidence for the existence of a direct interaction between Pol δ and the replisome (Figure 1B). Similar behaviors were observed in *in vivo* single-molecule experiments (Kapadia et al., 2020).

This linear template has already been used for single-molecule studies of protein roadblock encounters (Whinn et al., 2019; Schauer et al., 2020). However, there is currently no published work that uses single-molecule techniques to study lesion bypass. This is despite the development of the linear template to allow for the incorporation of site-specific lesions (Mueller et al., 2020; Kaur et al., 2022). While these site-specific lesions are not fluorescently-labeled and are therefore indistinguishable from an undamaged template in the absence of replication stalling, confirmation of a successful lesion incorporation method can be achieved through the insertion of a cy5 fluorophore at the same position using the same method (Figure 5E) (Kaur et al., 2022). It was shown that the *E. coli* replisome is efficiently stalled by an introduced CPD lesion (Figure 5F). In addition, the linear template can have lesions introduced in a non-specific manner through UV irradiation, where a dose of 1 J/m^2^ induces approximately one lesion per 100-kb (Bohr et al., 1985). These developments mean that the study of UV-induced DNA damage at the single-molecule level is now possible.

### Exchange dynamics during lesion bypass

In future, the application of these modifiable linear templates to a lesion bypass assay could provide valuable information regarding the dynamics of individual proteins during lesion bypass. Individual replisome proteins, as well as TLS polymerases can be labeled and visualized as lesion bypass takes place. Such an assay would allow the verification of the hypothesis that exchange dynamics are required for lesion bypass. Furthermore, the dependence of bypass efficiency on the rate of exchange can be visualized. The difference between leading-strand lesion bypass and lagging-strand lesion bypass can also be explored through these techniques. For example, it is tempting to speculate that the replicative helicase might pause upon encountering a lesion on the strand it is translocating on. While single-molecule fluorescence techniques can provide detailed temporal information and reveal stoichiometries within the replisome, the spatial resolution of these methods is often not sufficient to study the exact nature of interactions within the complex.

## Cryo-electron microscopy

### Cryo-electron microscopy provides structural information

Structural biology methods allow for the observation of macromolecules in order to gain insight into their three-dimensional structure, as well as how this structure reflects and influences function. Nuclear magnetic resonance (NMR) spectroscopy, X-ray crystallography, and cryo-electron microscopy (cryo-EM) are three of the main structural biology techniques, however cryo-EM is largely considered to be preferred technique due to recent advances (Herzik, 2020).

Single-particle cryo-EM data is obtained by applying a sample to a grid and then flash-freezing using liquid ethane, trapping the protein particles in ice. Flash freezing is required as it does not allow enough time for the formation of a crystalline lattice within the ice. Amorphous (non-crystalline) ice protects the sample and prevents the image being obscured. A beam of electrons are applied to the grid, resulting in an array of two-dimensional particle images, which can then be compiled computationally to yield a three-dimensional structure (Herzik, 2020).

Cryo-EM can be used to study the dynamics of DNA replication to an extent, but not in the same way as the single-molecule techniques, such as TIRF microscopy, described above. That is, you cannot visualize a single molecule undergoing DNA replication, nor can you visualize lesion encounter in real time using cryo-EM. Furthermore, resolving entire replisomes encountering a lesion is challenging due to the inherent flexibility and dynamics within a large multi-protein complex such as the replisome. This flexibility complicates the averaging of particles required to obtain high-resolution structures. Similar to ensemble methods, however, protein dynamics can sometimes be inferred.

In 2019, Jain et al. produced a near atomic resolution cryo-EM structure of yeast Pol δ holoenzyme in the act of DNA synthesis (Jain et al., 2019). This structure provided a framework for the understanding of DNA transactions at the fork. This is due to the structure revealing an unexpected arrangement consisting of the regulatory subunits, Pol 31 and Pol 32, lying next to the exonuclease domain of Pol 3 but not engaging the DNA. In addition, it was found that the catalytic and regulatory subunits of Pol δ rotate relative to each other, which is a key feature of Pol δ architecture. It can be seen that these structural features provide insight into DNA transaction mechanisms, despite not involving the real-time visualization of DNA replication and roadblock bypass.

When DNA is damaged, high-fidelity polymerases stall and PCNA is mono-ubiquitylated, which facilitates the recruitment and retention of TLS polymerases to the damaged sites *in vivo* and in a fully reconstituted yeast replisome. The structural basis of the interaction of TLS polymerases with both DNA and unmodified or mono-ubitquitylated PCNA, and therefore the mechanism of TLS polymerase recruitment to sites of DNA damage, remain poorly understood. In 2021, Lancey et al. (2021) identified this gap in knowledge and used cryo-EM to investigate.

The human TLS polymerase, Pol κ was the focus of this study. However, structural similarities are present between all TLS polymerases, and orthologs of Pol κ exist in bacteria and archaea, meaning that conclusions can be applied more widely than just human applications.

**FIGURE 6:**
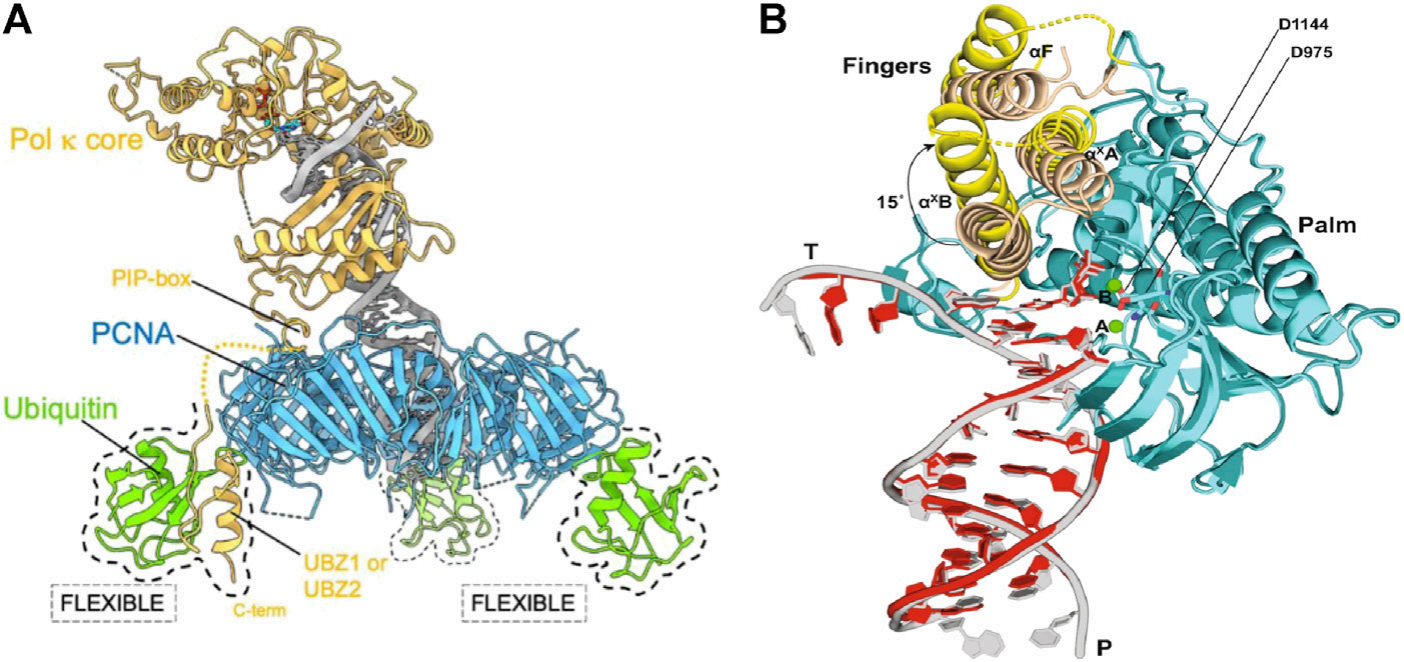
Cryo-EM structures suggesting flexibility of TLS polymerases **(A)** Proposed cryo-EM structure of the flexible binding of a TLS polymerase (Pol κ) to PCNA. Pol κ binds with mono-ubiquitylated PCNA through interacting with the PIP-box. Flexibility of ubiquitin molecules allows for interactions with Pol κ UBZ zinc fingers should the PIP-box interaction become compromised. This ensures that the TLS polymerase is retained to the DNA primer/template junction (Lancey et al., 2021). **(B)** Proposed cryo-EM structure of DNA-Pol ζ ternary complex. Fingers domain of Pol ζ on the mismatched template (yellow) adopts an open conformation, whereas the same domain on the matched template (wheat) is closed. An open conformation of the fingers domain in the presence of mismatched DNA allows for movement and accommodation of DNA lesions (Malik et al., 2022).

Cryo-EM reconstruction found indications of partial flexibility of ubiquitin moieties, as well as a flexible conformation of Pol κ bound to PCNA in the absence of DNA (Figure 6A). These findings aid in proposing a flexible structural framework that explain how PCNA recruits Y-family TLS polymerases to sites of DNA damage as they are required, despite these techniques not being able to visualize this recruitment in real time.

The eukaryotic TLS polymerase, Pol ζ is known to be more efficient than other TLS polymerases at extending DNA synthesis past DNA damage. However, the reason for this is largely unknown. In 2022, Malik et al. sort to gain insight into this higher efficiency through the investigation of the cryo-EM structure of *S. cerevisiae* Pol ζ (Malik et al., 2022) To do this, the structural differences of the DNA-Pol ζ ternary complex in the presence of matched and mismatched DNA was first investigated. While it was found that the Rev3, Rev7_A_, Rev7_B_, Pol 31, and Pol 32 subunits of Pol ζ were organized around the matched and mismatched duplex DNA in the same conformation, that is a pentametric ring-like structure, it was found that the fingers domain of Pol ζ on the mismatched template adopts an open conformation. In addition, the finger helices were less defined in the cryo-EM density when compared to that of the matched complex. It was also found that the replicative end of the mismatched DNA was less defined than that of the matched DNA. This indicates increased motion of the Pol ζ active site when the primer terminus contains a mismatch. Together, this suggests that the open conformation in the presence of mismatched DNA allows for movement around that conformation, and the resultant extra space allows for the accommodation of bulky DNA lesions and mismatches (Figure 6B) (Malik et al., 2022).

From the results of this study, it was hypothesized that Pol ζ’s heightened efficiency at extending DNA synthesis past DNA damage comes as a result of Pol ζ’s lack of proofreading exonuclease activity and the overall flexibility of the structure, as well as the absence of a β-hairpin structure which, in B-family polymerase exonuclease domains, is thought to facilitate the transfer of a mismatched primer from the polymerase to the exonuclease active site. These factors cause Pol ζ to differ from other B-family polymerases, and can be thought to preferably promote the extension reaction in the presence of mismatched DNA (Malik et al., 2022).

### Structural intermediates during lesion bypass

From the cryo-EM studies discussed here, it can be seen that structural information, while not dynamic in nature, can still provide insight into the dynamics of individual proteins and replisomal components. Through the identification of flexible conformations and ternary complexes, the recruitment of TLS polymerases can be further understood, with insight gained that single-molecule techniques such as TIRF microscopy can not alone provide. However, thus far, cryo-EM has not been used to study the dynamic binding and unbinding of proteins within the replisome upon lesion encounter. Structures from such studies would provide important information on the nature and number of protein binding sites. Furthermore, structural information could reveal the decoupling of the helicase from the polymerases upon lesion encounter.

## Bridging the gap between static structures and dynamics

Many complex biological processes are hard to decipher using just one technique. Instead, pieces of information can be gathered from multiple individual techniques and then this information can be compiled in order to model a functional biological system (Banerjee et al., 2021). This highlights the importance of bridging the gap between cryo-EM and single-molecule studies, to gain a complete understanding of the observable dynamics of the replisome during lesion bypass, as well as the structures that make these dynamics possible. In order to understand the molecular mechanisms of biological processes, short-lived intermediate states of pathways and processes must be structurally deciphered.

Recent developments in cryo-EM have allowed for the visualization of sequential steps and intermediates in biochemical pathways, helping to bridge the gap between the “static” approach of traditional cryo-EM, and the dynamic single-molecule fluorescence imaging approaches. These developments include time-resolved cryo-EM, and *in silico* reconstitution of DNA replication applied to single-particle cryo-EM (Miller et al., 2019).

Time-resolved cryo-EM can be used to image short-lived intermediate states of biological processes by trapping transient conformational changes. This is done through vitrification, which is the grid preparation process, at specific time points following the initiation of the reaction (Dandey et al., 2020). The advantages and value of time-resolved cryo-EM have been demonstrated through the applications of ribosomal subunit binding, the binding of promoter DNA to RNA polymerase (RNAP), binding of calcium to a potassium channel inducing a conformational change, and conformational rearrangements of dynamin lipid tubes driven by GTP hydrolysis. Dandey et al. (2020) found that this technique successfully provided insight into conformational changes during these biological processes, however it was noted that further work was required to determine whether the vitrification process altered kinetics and thermodynamics.

In addition to time-resolved cryo-EM, *in silico* reconstitution of single-particle cryo-EM data can also be used to describe complex dynamic systems (Miller et al., 2019; Greiwe et al., 2022). Similar to other structure determination methods, *in silico* reconstruction of single-particle cryo-EM first involves picking particles and extensive two-dimensional averaging. Then, the structural averages are positioned back into the original image that the raw particles were cropped out of using coordinates derived from particle picking and any rotations that were applied during two-dimensional classification. This technique is therefore specifically useful for the study of proteins within their macromolecular environment (Miller et al., 2019; Greiwe et al., 2022; Lewis et al., 2022).

Another emerging technique that is bridging the gap between cryo-EM and fluorescence microscopy is correlative light EM (CLEM). CLEM works by combining fluorescence microscopy and cryo-EM in two sequential steps. First, fluorescence microscopy is used to inspect the sample and identify notable features and dynamic events. The location of these features is then used to direct the inspection of the same sample by cryo-EM, allowing for high resolution structural information of these areas of interest to be obtained (Sartori et al., 2007; Tuijtel et al., 2019). While CLEM is yet to be applied to the study of lesion bypass and replisome dynamics, the technology presents a promising way to obtain both structural and dynamic information from the same sample, whilst also presenting an efficient way of directing the focus for cryo-EM.

Finally, the development of structure prediction algorithms such as AlphaFold (Jumper et al., 2021; Kryshtafovych et al., 2021) and RoseTTAFold (Baek et al., 2021), provides another emerging pathway to combine structural information with dynamics. These structure prediction programs, based on deep-learning algorithms, predict protein structure with astonishing accuracy. By mid 2022, structures for nearly all catalogued proteins had been predicted using these algorithms. Structure prediction of very large multi-protein complexes, such as the replisome, is not possible yet. Furthermore, the interactions of nucleic acids with these complexes can not yet be solved through prediction. Therefore, a combination of structure determination and structure prediction tools, along with single-molecule studies, could provide a tremendously powerful approach to solving multi-protein complex structures.

The techniques described here have thus far not been applied to the study of replisome-lesion encounter. However, all four techniques present exciting and promising developments that may help bridge the gap between the static high-resolution structural information provided by cryo-EM, and the dynamic information provided by single-molecule techniques. By resolving the structures of transient states that may not yet be documented and combining these structures with temporal information obtained from single-molecule studies, entire molecular pathways could be elucidated.

## Conclusion and outlook

This review focused on biochemical and biophysical methods that can be used to study protein dynamics during replisome–lesion encounters. Specifically we discussed ensemble biochemical assays, single-molecule visualization methods, and cryo-EM.

Ensemble averaging methods are well-established, relatively high-throughput techniques that can be used to test many different reaction conditions in a short amount of time at comparatively low cost. By cleverly designing the assays, protein dynamics can be inferred from the experimental outcome. However, while dynamics and structural information can be inferred, they cannot be observed. Cryo-EM and single-molecule techniques provide more direct access to the dynamic behavior of proteins. Besides giving high-resolution spatial information, cryo-EM can now also provide information on the changes in composition of multi-protein complexes during a biochemical reaction. Using time-resolved cryo-EM methods structures of complexes can be solved at several stages in the reaction. However, the exact time spent at each stage and the exact dynamic pathways are hard to determine. Single-molecule techniques on the other hand do not have the high spatial resolution of cryo-EM but provide a higher temporal resolution. Furthermore, single-molecule assays can pick up on short-lived intermediates and rare events that might be hard to capture in cryo-EM. Herein lies an immediate opportunity to combine the strengths of both cryo-EM and single-molecule techniques. The changes in structures of multi-protein complexes observed in cryo-EM can be linked temporally through single-molecule observations.

The developments in the single-molecule techniques have allowed for the observation and measurement of replisomal dynamics *in vitro*. This includes visualizing DNA replication in real time in both the presence and absence of DNA damage using TIRF microscopy. These imaging methods can include the fluorescent labeling of individual replisome components in order to visualize their individual dynamics (Leisle et al., 2016). However, this development is yet to be applied to the visualization of lesion bypass and its associated dynamics, despite creating the foundations to allow for such a study. An immediate use for this application lies in the visualization of SSBs. As many lesion bypass mechanisms result in a ssDNA region being left behind at the site of the lesion, highlighting this ssDNA region would theoretically allow for the confirmation that lesion has bypass occurred. In TIRF microscopy, using fluorescently-labeled SSBs in place of unlabeled SSBs would allow for the visualization of SSBs binding to the ssDNA regions in the wake of the replisome following lesion bypass. ssDNA regions are generally very flexible, even when bound by SSBs, making their detection by cryo-EM less practical.

The observations using techniques presented in this review paint a very dynamic picture of replisome-lesion bypass. There are multiple molecular pathways available to the replisome upon encountering the lesion. The pathway(s) that are used depend on many factors including timing, the strand the lesion is on, and the availability of accessory factors to the replisome. Furthermore, the inherent stochastic behavior of the replisome provides it with the opportunity to use multiple pathways for the same situation. The helicase seems to be the most stably bound replisome protein, both in prokaryotes as well as eukaryotes. Helicases can accommodate most chemical lesions and can unwind past the lesion. It is therefore hypothesized that the helicase forms the stable platform that allows all other replisome proteins to dynamically bind to (Spinks et al., 2021). Lesion skipping, whereby the replisome reassembles and replication reinitiates, is a pathway that is available to both prokaryotes and eukaryotes. This pathway critically depends on the availability of replisome components in solution to exchange into the replisome. The concentration of these proteins in cells is high enough, to ensure availability of this pathway. TLS polymerases can get access to the replisome through exchange with the replicative polymerases. However, replicative TLS does not seem to be the predominant pathway. More commonly, TLS polymerases act on lesions in the wake of the replisome.

Throughout this review, we have focused on UV-induced DNA lesions as a roadblock to DNA replication. This emphasis is largely informed by the prevalence and relevance of UV-induced DNA lesions. However, the methods outlined here can be applied to the study of other chemically induced lesions. Furthermore, the similar methodology can be used to study replisome encounters with other roadblocks such as secondary structures and protein complexes. Secondary structures such as G-quadruplexes have been known to slow replication, and can be further stabilized to form a more stable block (Alessandrini et al., 2021; Kosiol et al., 2021). Key examples of relevant protein complexes include cohesin (Rhodes et al., 2017), nucleosomes (Dequeker et al., 2022), and (stalled) transcription complexes (Pomerantz and O’Donnell, 2010; McGlynn et al., 2012; Lang et al., 2017). Given the relevance of all of these roadblocks in human disease pathways, we see many opportunities to study replisome-roadblock encounters in the near future.
